# Pedigree and genome-based patterns of homozygosity in the South African Ayrshire, Holstein, and Jersey breeds

**DOI:** 10.3389/fgene.2023.1136078

**Published:** 2023-03-17

**Authors:** Carina Visser, Simon Frederick Lashmar, Jason Reding, Donagh P. Berry, Esté van Marle-Köster

**Affiliations:** ^1^ Department of Animal Science, Faculty of Natural and Agricultural Sciences, University of Pretoria, Pretoria, South Africa; ^2^ Animal and Grassland Research and Innovation Centre, Teagasc, Co. Cork, Ireland

**Keywords:** autozygosity, cattle, inbreeding, pedigree, runs of homozigosity, single nucleotide polymorphism

## Abstract

The erosion of genetic diversity limits long-term genetic gain and impedes the sustainability of livestock production. In the South African (SA) dairy industry, the major commercial dairy breeds have been applying estimated breeding values (EBVs) and/or have been participating in Multiple Across Country Evaluations (MACE). The transition to genomic estimated breeding values (GEBVs) in selection strategies requires monitoring of the genetic diversity and inbreeding of current genotyped animals, especially considering the comparatively small population sizes of global dairy breeds in SA. This study aimed to perform a homozygosity-based evaluation of the SA Ayrshire (AYR), Holstein (HST), and Jersey (JER) dairy cattle breeds. Three sources of information, namely 1) single nucleotide polymorphism (SNP) genotypes (3,199 animals genotyped for 35,572 SNPs) 2) pedigree records (7,885 AYR; 28,391 HST; 18,755 JER), and 3) identified runs of homozygosity (ROH) segments were used to quantify inbreeding related parameters. The lowest pedigree completeness was for the HST population reducing from a value of 0.990 to 0.186 for generation depths of one to six. Across all breeds, 46.7% of the detected ROH were between 4 megabase pairs (Mb) and 8 Mb in length. Two conserved homozygous haplotypes were identified in more than 70% of the JER population on *Bos taurus* autosome (BTA) 7. The JER breed displayed the highest level of inbreeding across all inbreeding coefficients. The mean (± standard deviation) pedigree-based inbreeding coefficient (F_PED_) ranged from 0.051 (±0.020) for AYR to 0.062 (±0.027) for JER, whereas SNP-based inbreeding coefficients (F_SNP_) ranged from 0.020 (HST) to 0.190 (JER) and ROH-based inbreeding coefficients, considering all ROH segment coverage (F_ROH_), ranged from 0.053 (AYR) to 0.085 (JER). Within-breed Spearman correlations between pedigree-based and genome-based estimates ranged from weak (AYR: 0.132 between FPED and F_ROH_ calculated for ROH <4Mb in size) to moderate (HST: 0.584 between F_PED_ and F_SNP_). Correlations strengthened between F_PED_ and F_ROH_ as the ROH length category was considered lengthened, suggesting a dependency on breed-specific pedigree depth. The genomic homozygosity-based parameters studied proved useful in investigating the current inbreeding status of reference populations genotyped to implement genomic selection in the three most prominent South African dairy cattle breeds.

## 1 Introduction

The early migration of cattle to Southern Africa and the introduction of exotic cattle into the native landscape are still debated (Orton et al., 2013). Although it is commonly believed that dairy cattle were first introduced to South Africa (SA) by the Dutch East India Company (VOC) during the 17^th^ century to supply fresh milk to crews of ships passing the Cape of Good Hope, Felius et al. (2014) reported that the first Friesian cattle were only introduced to South Africa in 1850. The first Holstein-Friesian was registered in 1906 with SA Stud Book, and the Breeders’ Society was founded in 1912 (Duvenhage, 2017).

The years 1881 and 1890 have been reported as the most probable dates for the arrival of the first Jersey and Ayrshire cattle in South Africa, respectively (SA Stud Book, 2004). The Ayrshire Cattle Breeders’ Society of South Africa was established in 1916, followed by the South African Jersey Cattle Breeders’ Society in 1920 (Nel, 1968). It can, therefore, be concluded that European dairy cattle have been farmed in South Africa for more than a century. No additional breed development was performed in South Africa, apart from normal selection practices. The SA dairy populations have strong international genetic linkage due to extensive use of artificial insemination. The composition of the breeding objectives is similar to those of other international populations (Cole & VanRaden, 2018).

Official animal recording for dairy cattle in South Africa dates back to 1917, with the inception of a milk recording scheme (Van Marle-Köster and Visser, 2018), which was developed over time to include contemporary comparison methods in the 1970s, followed by the implementation of the Best Linear Unbiased Prediction (BLUP) sire model in 1987 (Mostert et al., 2004). Since the early 1990s, routine genetic evaluations have provided the Ayrshire (AYR), Holstein (HST), and Jersey (JER) breeders with estimated breeding values (EBVs) to be used in selection decisions. Routine participation in INTERBULL for Multiple Across Country Evaluations followed in 2004 (Mostert et al., 2006).

The breeding objectives for all three breeds include milk yield, milk quality, fertility, and functional traits (Banga et al., 2014; SA Stud Book, 2022a; SA Stud Book, 2022b; SA Stud Book, 2022c). More recently, funding initiatives such as the Dairy Genomics Program (DGP) have facilitated the establishment of single nucleotide polymorphism (SNP)-genotyped reference populations to help generate genomic breeding values for these breeds (Van der Westhuizen and Mostert, 2020).

Inbreeding results from consanguineous mating inevitably leading to an increased frequency of homozygosity. The phenomenon of reduced performance due to inbreeding known as inbreeding depression has been reported for a plethora of traits in dairy cows including milk production, reproduction, and fitness (e.g., survival), thereby impacting overall herd profitability (Doekes et al., 2019; Makanjuola et al., 2021). Historically, inbreeding was measured using pedigree information, based on the calculation of the probability that an individual has inherited alleles identical by descent (Wright, 1978). This measure of inbreeding is, however, dependent on both accurate and deep ancestry records (Ablondi et al., 2022; Saif-ur-Rehman et al., 2022). With the growing availability of genome-wide genotype information on large populations of animals, genome-based estimates of inbreeding are replacing pedigree-based estimates as the statistics of choice.

Runs of homozygosity (ROH) segments are detected using genome-wide genotype information with the profiling of these segments providing a well-established methodology to quantify genetic autozygosity and genetic diversity (Gautason et al., 2021; Ablondi et al., 2022; Mulim et al., 2022). Detected ROH are identifiable as continuous segments of homozygous nucleotide sequences that are highly correlated with mutation loads (Makanjuola et al., 2021) and can be indicative of the age of inbreeding based on their length characteristics (Gautason et al., 2021). Additionally, shared ROH segments, harboring SNP haplotypes that have a higher incidence compared to a certain population-specific threshold (Gorssen et al., 2021), may help guide the localization and/or identification of chromosomal regions under artificial or natural selection.

The widespread use of certain local and international bulls may contribute to greater genome-wide and location-specific homozygosity with downstream repercussions on productivity. In the South African dairy industry, the majority of bull semen used is of foreign origin with more than 40% of Holstein semen imported from the United States of America (USA); Canadian bloodlines predominate in the Ayrshire bulls used in South Africa while most Jersey bulls used in South Africa are of USA origin. Semen from countries such as Great Britain, Denmark, France, the Netherlands, Australia, and New Zealand have also contributed to the South African dairy cattle gene pool (Opoola et al., 2020). The ancestral information of these sires is available through Interbull; the depth of pedigree available is, however, dependent on each participating organization (International Bull Evaluation Service—INTERBULL, 2022). South Africa, with a relatively small dairy population compared to many other countries, needs to be able to manage the extent of genetic diversity within its dairy sector. Having access to genome-wide genotype information on individual animals provides an opportunity to evaluate the genetic diversity and inbreeding of the local South African dairy populations.

The objectives of the present study were to 1) classify and quantify runs of homozygosity in three South African dairy cattle populations; 2) estimate inbreeding coefficients using various sources of information, and 3) compare the inbreeding statistics generated from either recorded ancestry or genomic information.

## 2 Materials and methods

Ethical approval was granted by the University of Pretoria’s Ethics Committee for external data use (EC170627-135). Consent was provided from the respective breeders’ societies to allow access to the available pedigree and genotypic data.

### 2.1 Pedigree data

Pedigree data of the genotyped South African Ayrshire (AYR), Holstein (HST), and Jersey (JER) populations used in the present study were provided by SA Stud Book. The pedigree information included 7,885 AYR (5,654 females, 2,231 males), 28,391 HST (20,921 females, 7,470 males), and 18,755 JER (14,138 females, 4,617 males) records as summarized in Table 1. The pedigree depth was up to 24, 30, and 26 generations deep for the genotyped AYR, HST, and JER breeds, respectively. The optiSel (Wellmann and Bennewitz, 2019) R package was utilized to calculate the complete generation equivalent (CGE) and the pedigree completeness index (PCI) for each of the individual genotyped animals.

**TABLE 1:** A summary of the number of animals included in the pedigree analyses and genomic analyses for the Ayrshire (AYR), Holstein (HST), and Jersey (JER) breeds.

Population	Pedigree analyses	Birth year range	Genomic analyses	Birth year range
AYR	7,885	1910–2017	510	1973–2017
HST	28,391	1917–2021	1,360	1981–2021
JER	18,755	1931–2021	1,329	1989–2021
Total	55,031		3,199	

### 2.2 Single nucleotide polymorphism (SNP) genotypic data and quality control

A total of 3,199 genotyped animals (2,732 female, and 467 male cattle) with a sample call rate above 95% were available for this study consisting of 510 AYR, 1,360 HST, and 1,329 JER cattle. The animals included in this study originated from the national Dairy Genomic Program (DGP) with the aim of establishing reference populations for genomic selection. Animals that were included in this program represented the local populations and were selected based on EBV accuracies of at least 60%. The data structure of the genotyped populations is summarized in Table 1. The year of birth of genotyped animals ranged from 1973 to 2017 for the AYR, 1981 to 2021 for the HST, and 1989 to 2021 for the JER. For pedigree depth and inbreeding estimates, only the pedigree of the genotyped animals was considered.

All AYR animals were genotyped using the BovineSNP50-24 version 3 (Illumina, Inc. San Diego, CA 92122 USA) array containing 53,218 SNPs. Genotypes of HST and JER animals originated from five different genotyping panels, namely, the Bovine SNP50 versions 1 (54,001 SNPs) and 3 (53,218 SNPs), GeneSeek^®^ Genomic Profiler™ 150K (139,480 SNPs), International Dairy and Beef (IDB) version 3 (53,450 SNPs), Weatherbys Scientific VersaSNP 50K™ (49,788 SNPs), and the Unistel-SA Stud Book 50K version 1 (54,394 SNPs) panels. The GeneSeek^®^ Genomic Profiler™ 150K genotypes were generated through the South African DGP that was initiated in 2016 to benefit herds that participate in pedigree-based genetic evaluations and/or milk recording schemes provided by the Agricultural Research Council (ARC) or SA Stud Book. Genotype calling was done through various local and international service providers using their respective protocols, and the raw genotype files were converted into PLINK software version 1.9 (Purcell et al., 2007) input files. A common set of 36,887 SNPs were extracted for each population and the data sets were merged for the across-population analyses.

Sample- and marker-based quality control edits were performed using PLINK software version 1.9 (Purcell et al., 2007) to filter out non-autosomal and low-quality (SNP call rate<95%) SNPs from the dataset. As suggested by Meyermans et al. (2020) for ROH detection, neither minor allele frequency (MAF) nor linkage disequilibrium (LD) filtering was applied. No SNP edits were performed based on Hardy-Weinberg Equilibrium (HWE). The post-editing data set consisted of 3,199 animals with 35,572 autosomal SNP genotypes and all subsequent analyses were undertaken using this data set. The same animals were thus used for the pedigree and genomic analyses.

### 2.3 Genomic relatedness

GCTA version 1.24 (Genome-wide Complex Trait Analysis; Yang et al., 2011) was used to estimate genetic relatedness between individuals from the set of 35,572 autosomal genome-wide SNPs. A genomic relationship matrix was calculated using the method by Yang et al. (2010) and was followed by the estimation of eigenvalues and eigenvectors for a principal component analysis (PCA). The eigenvectors per animal were plotted as a scatter plot to visualize genomic relatedness.

### 2.4 Runs of homozygosity detection

Runs of homozygosity (ROH) for all genotyped animals were detected using the R package *detectRUNS* (Biscarini et al., 2019) by executing both the consecutive-SNP-based detection method (CR) and sliding window approach (SW; Marras et al., 2015). The SW approach, and more specifically its application in PLINK software (Purcell et al., 2007), is generally the most common ROH detection approach (and, hence, resource for F_ROH_ estimation) used across all livestock species (Peripolli et al., 2017) and has previously proven to outperform other methods (e.g., Howrigan et al., 2011). Dixit et al. (2020), for example, reported similar results for the *detectRUNS* SW approach to that of PLINK. The CR algorithm, which executes a window-free SNP-by-SNP approach, has received less research attention, however, has previously been shown to produce F_ROH_ patterns similar to that of both PLINK and *detectRUNS*’ SW approaches despite discrepancies in the number of ROH identified (Dixit et al., 2020). Both approaches were, therefore, tested in this study for a more comprehensive profiling of ROH.

For CR, the following ROH defining parameters were set: i) a minimum length of 1Mb, ii) a maximum distance (gap) between consecutive SNPs of 500kb, iii) a lower density limit of one SNP per 75kb, and iv) a maximum of two missing and no opposing (heterozygous) genotypes were allowed. The aforementioned parameters were the same for the SW approach, but, the sliding window size was set to 50 SNPs. The minimum number of SNP that constituted an ROH segment was set to 54 based on the formula implemented by Purfield et al. (2012):

$$l=\frac{\log_{e\frac{\alpha }{n_{s}.n_{i}}}}{\log_{e}\left(1-\bar{het}\right)}$$

where *n*_*s*_ and *n*_*i*_ were the numbers of SNPs and individuals, respectively, α represented the proportion of false-positive identifications (set to 0.05) and $\bar{het}$ was the average SNP heterozygosity. The detected ROH were assigned to one of four length categories: <4 Mb, 4≤ROH<8 Mb, 8≤ROH<16 Mb, or ≥16 Mb. The *detectRUNS* (Biscarini et al., 2019) package was additionally used to obtain the proportion of times each SNP fell inside an ROH within each population. Based on the produced Manhattan plots, ROH regions identified in >75% of the JER population, and >25% in the AYR and HST populations were investigated using the Ensemble BioMart online tool (http://asia.ensembl.org/biomart/martview/244b07db6f169a19f1e0362778df6ab5). Gene ontology and pathway analyses were carried out by PANTHER version 13.1 software tool (http://pantherdb.org).

### 2.5 Inbreeding coefficients

Three methods were used to estimate the inbreeding coefficients of all genotyped individuals: 1) F_PED_ represented a pedigree-derived estimate, 2) F_SNP_ represented an SNP-by-SNP excess in homozygosity, and 3) F_ROH_ represented genome-wide ROH coverage. The F_PED_ and F_SNP_ coefficients were calculated using optiSel (Wellmann and Bennewitz, 2019) and PLINK software version 1.9 (Purcell et al., 2007), respectively. The F_PED_ coefficient was calculated using the *summary.Pedig* function in optiSel, which estimates the inbreeding coefficient as defined by Meuwissen and Luo (1992). For F_SNP_, the --het function in PLINK was executed, which is based on the formula $F_{SNP}=\frac{O-E}{N-E}$ , where *O* is the observed number of homozygous SNPs per individual, *E* is the expected number of homozygous SNPs under the Hardy-Weinberg equilibrium (HWE) calculated based on the estimated allele frequencies of the sample, and *N* is the total number of SNPs. Additionally, the observed as well as expected heterozygosity rates (H_O_, and H_E_, respectively) were estimated as the total number of non-missing genotypes (NNM) minus the number of observed homozygous genotypes (HOM) divided by the total non-missing genotypes (NNM).

All F_ROH_ coefficients were based on the ROH detected with the SW approach for comparability. The F_ROH_ coefficient was estimated as (McQuillan et al., 2008):

$$F_{ROH}=\frac{\Sigma L_{ROH}}{\Sigma L_{AUTO}}$$

where L_ROH_ represented the length of ROH in one individual, and L_AUTO_ represented the length of the genome covered by SNPs, excluding the centromeres. Separate F_ROH_ coefficients were additionally calculated based on the length categories previously described and were labeled $F_{{ROH}_{<4Mb}}$ , $F_{{ROH}_{4\leq x<8Mb}}$ , $F_{{ROH}_{8\leq x<16Mb}}$ , and $F_{{ROH}_{\geq 16Mb}}$ . Comparisons between these statistics were made by means of Spearman rank (rho) correlations calculated within-breed using the cor.test function in R software (R Core Team, 2013).

### 2.6 Effective population size

The effective population size (Ne) of an actual population can be defined as the size of a hypothetical ideal population resulting in the same amount of genetic diversity as is present in the real population (Wright, 1978). The Ne based on both pedigree and SNP data were estimated separately. The estimated Ne based solely on pedigree information is limited by the pedigree depth (and accuracy of recording), whereas the SNP-based method is able to estimate both historical and recent Ne but is limited by the extent of LD captured (and hence, the SNP genotyping panel density as well as the number of animals genotyped). The pedigree-based Ne was calculated using the optiSel (Wellmann and Bennewitz, 2019) R package (R Core Team, 2013) for the last 10 complete generations. The SNP-based estimates of historical (highest number of generations ago) and recent Ne (least number of generations ago) were calculated using SNeP v.1.1 software (Barbato et al., 2015) based on linkage disequilibrium (LD) and by implementing the approximation proposed by Sved (1971) as a recombination rate modifier.

## 3 Results

### 3.1 Pedigree completeness and pedigree-based population structure

The mean, interquartile range (IQR), and median years of birth for the genotyped AYR population was 1974, 1956 to 1994, and 1972, respectively; 1973, 1953 to 1992, and 1969 for the genotyped HST population, respectively and 1980, 1960 to 1998, and 1982 for the JER population, respectively. The mean pedigree completeness index (PCI) of the genotyped populations was 0.976 for the AYR, 0.967 for the HST, and 0.993 for the JER populations. The average pedigree depth based on CGE was equal to 9.75 for AYR, 11.70 for HST, and 10.05 for JER.

The mean six-generation deep pedigree completeness for genotyped animals born in the 10-year period between 2011 and 2021 for the HST and JER breeds, and between 2007 and 2017 for the AYR breed is summarized in Table 2. These animals represented between 9% and 11% of the fully-traced back pedigree of the genotyped populations. The genotyped HST breed consistently showed the lowest pedigree completeness from six to two generations ago at 0.186 to 0.544 while the genotyped AYR (0.288–0.702) and genotyped JER (0.278–0.682) breeds had similar pedigree completeness six to two generations ago.

**TABLE 2:** The mean six-generation deep pedigree completeness for the genotyped South African Holstein (HST) and Jersey (JER) animals born within the period 2011 to 2021 as well as Ayrshire (AYR) animals born within the period 2007 to 2017.

	Generation depth					
Population	1	2	3	4	5	6
AYR	1.000	0.702	0.523	0.414	0.341	0.288
HST	0.990	0.544	0.369	0.278	0.223	0.186
JER	1.000	0.682	0.507	0.399	0.329	0.278

### 3.2 Genome-based genetic relatedness

**FIGURE 1:**
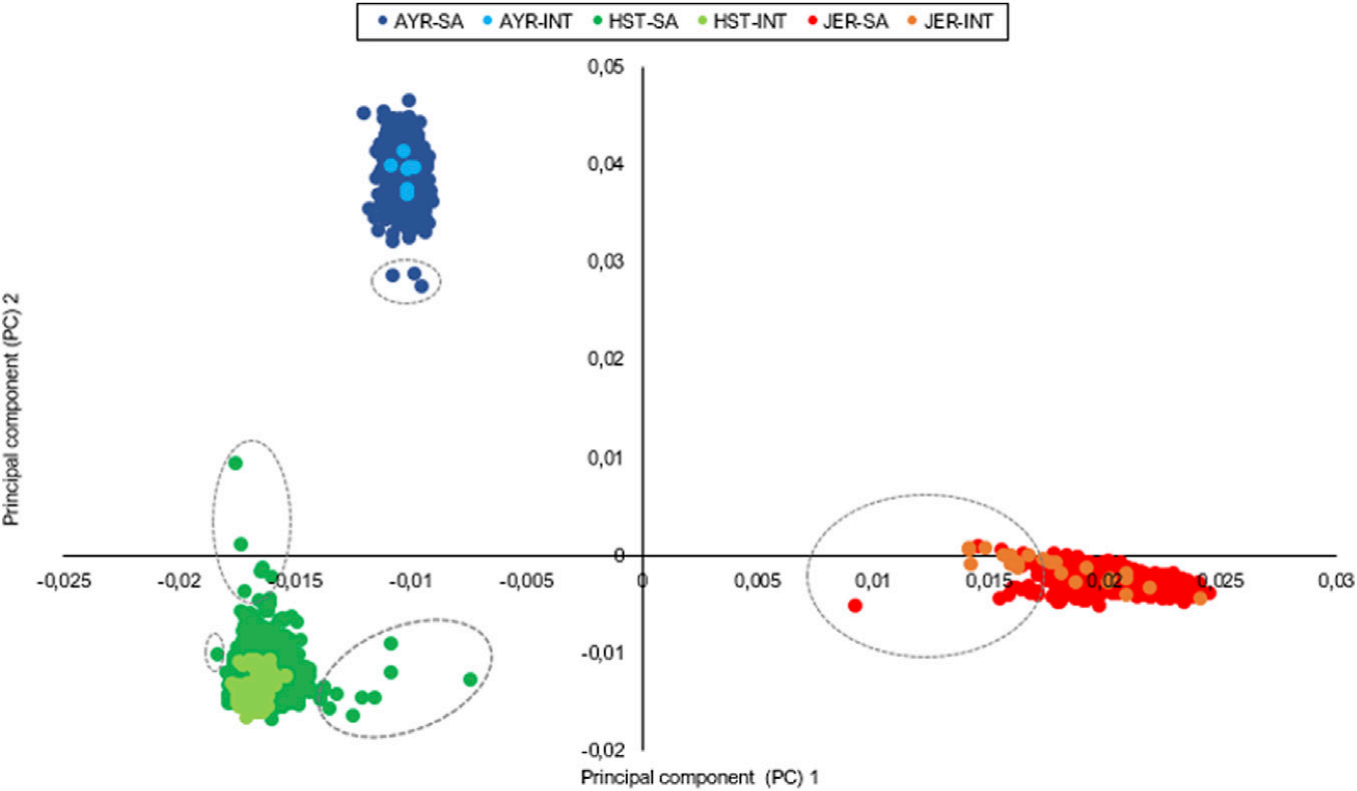
Principal components illustrate the genetic relatedness between and within the sampled Ayrshire (AYR), Holstein (HST), and Jersey (JER) populations with outliers encircled. INT, international animals; SA, South African animals.

The first and second principal components of the autosomal SNP genotypes explained 8.3% and 4.6% of the genetic variation between all individuals and grouped the animals into three distinct clusters that corresponded with the three separate breeds. For the first principal component, the standard deviation of the eigenvectors ranged from 0.4 × 10^−3^ for AYR to 0.002 for JER (Figure 1). The number of outliers (encircled with gray dotted lines in Figure 1), defined as animals with eigenvectors outside the boundaries of mean ± 3 standard deviations for the first and/or second principal components, were three, 15, and 11 for the AYR, HST, and JER populations, respectively. While all identified outliers in the AYR and HST populations were South African animals, five of the JER outliers were international bulls (two from New Zealand and three from Denmark).

### 3.3 Identified runs of homozygosity (ROH)

The per-breed statistics of the identified ROH are summarized in Table 3. Irrespective of breed, the CR ROH detection method identified more homozygous runs compared to the SW approach. For both detection methods, the majority of detected ROH was in the JER, followed by the HST and AYR breeds. The mean (±standard deviation) per individual ROH counts was 17.99 ± 4.96, 16.67 ± 5.47, and 28.30 ± 6.30 for the AYR, HST, and JER populations, respectively when the SW ROH detection approach was employed (CR method: AYR = 25.05 ± 6.89, HST = 23.70 ± 7.57, JER = 39.54 ± 8.32). The mean (±standard deviation) length ROH detected was the largest for the HST population (SW method: 8.66 Mb ± 6.82 Mb) and the smallest for the AYR populations (SW method: 7.69 Mb ± 5.99 Mb). However, the mean (±standard deviation) genome-wide ROH coverage (i.e., the total length of the genome covered by ROH) was the greatest for JER (SW method: 221.15 Mb ± 69.27 Mb) and the lowest for AYR (SW method: 7.69 Mb ± 5.99 Mb).

**TABLE 3:** Summary statistics of runs of homozygosity (ROH) identified for the Ayrshire (AYR), Holstein (HST), and Jersey (JER) dairy breeds using two ROH detection methods.

	Breed	*n*ROH^c^	Mean_Total Length_ (Mb)^d^	Median_Total Length_ (Mb)^d^	Mean_ROH Length_ (Mb)^5^	Min_ROH Length (Mb) _(BTA)^e^	Max_ROH Length (Mb)_ (BTA)^e^
CR^a^	AYR	12,777	188.66	180.26	7.53	1.02 (BTA2)	48.21 (BTA8)
	HST	32,226	193.21	184.32	8.15	1.00 (BTA5)	69.49 (BTA6)
	JER	52,553	297.75	292.84	7.53	1.00 (BTA18)	76.59 (BTA4)
SW^b^	AYR	9,176	138.31	133.60	7.69	1.07 (BTA2)	63.45 (BTA6)
	HST	22,674	144.46	136.37	8.66	1.00 (BTA5)	75.54 (BTA6)
	JER	37,617	221.15	215.26	7.81	1.00 (BTA18)	90.92 (BTA4)

^a^ CR = consecutive SNP-based method.

^b^ SW = sliding window approach.

^c^ *n*ROH = number of ROH segments identified.

^d^ The mean and median of the summed ROH length (i.e., total ROH genome coverage) per individual.

^e^ The mean, minimum and maximum ROH length considering all individual ROH segments; BTA = *Bos taurus* autosome.

The autosome-wide distribution of the total number of ROH as well as the percentage coverage per autosome is illustrated in Supplementary Material S1. For all breeds, the most ROH were detected on BTA1 (range: 708 ROH for AYR to 3,475 ROH for JER), which is the largest autosome (158.2 Mb), whilst the fewest ROH (range: 110 for AYR to 440 for JER) were detected on BTA28 (46.2 Mb). For all breeds, the percentage of ROH coverage showed an increasing trend towards smaller autosomes (line graph in Supplementary Material S1) and peaked for BTA25, with values of 20.8%, 17.2%, and 16.3% estimated for the AYR, HST, and JER breeds, respectively. The lowest overall percentage autosomal coverage was observed for BTA5 (across all breeds: 6.84%).

**FIGURE 2:**
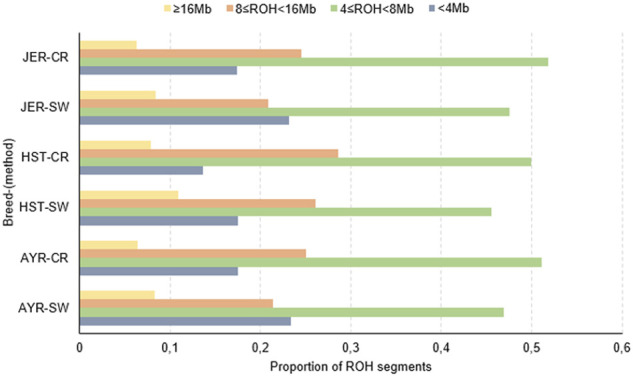
The proportions of all detected runs of homozygosity (ROH) in different length categories for the Ayrshire (AYR), Holstein (HST), and Jersey (JER) dairy breeds. CR, consecutive SNP-based method; SW, sliding window approach.

Across all breeds, and for both detection methods, the majority of detected ROH were within the 4 ≤ ROH<8 Mb length category. The distribution of ROH within different length (in Mb) categories is depicted in Figure 2. Despite the variation in the number of ROH identified per breed (e.g., SW method: 13,498 more ROH for HST compared to AYR and 14,943 more segments for JER compared to HST), the differences in the number per length category were negligible between AYR and JER. In comparison to the other breeds, the HST breed had a greater number of large (≥16 Mb) ROH identified by both detection methods (CR: 0.079; SW: 0.109).

**FIGURE 3:**
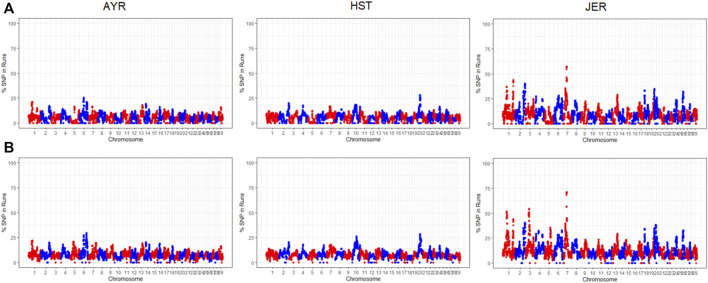
The chromosome-wide proportion of times each SNP resided within a detected ROH for the Ayrshire (AYR), Holstein (HST), and Jersey (JER) breeds using both the sliding window **(A)** and consecutive SNP-based **(B)** detection methods.

The proportion of times an SNP resided within a detected ROH was estimated per population. Two ROH haplotypes on BTA7 were identified in 70.96% of the JER population. The first preserved region consisted of 20 SNPs spanning 1.19 Mb and the second of 31 SNPs spanning 2.60 Mb. The first region encompasses 35 protein-coding genes, including *LYPD8* (Gram-negative bacteria defense response) and various olfactory receptor genes, whilst the second region encompassed 82 protein-coding genes, including *HSPA4* (heat-shock protein), *ELANE* (immune response), and *LEAP2* (antimicrobial humoral immune response). Manhattan plots of the incidence of each SNP within detected ROH per breed are illustrated in Figure 3. For the AYR breed, the highest occurring consensus ROH haplotype was on BTA6 (base pairs position: 90,665,860-90,902,316) in 28.82% of the population. The 0.236 Mb AYR region contains seven protein-coding genes, including the *PPEF2* (Hsp90 protein binding), as well as the *CXCL9* and *CXCL10* (both antimicrobial humoral immune responses). Three smaller ROH haplotypes, close in proximity, were identified on BTA20 (base pair position ranges: 38,453,649–38,487,130, 38,578,200–39,046,015, and 38,761,711–38,920,878) in 28.31%, 28.16%, and 28.09% of the HST population, respectively. These 0.054Mb, 0.181Mb, and 0.112 Mb regions contained two, four, and three SNPs, respectively. The 0.181 Mb genomic region overlaps with the *SPEF2* (sperm flagella 2 protein) protein-coding gene, whereas the 0.112 Mb overlaps with *PRLR*, a prolactin receptor.

### 3.4 Inbreeding coefficients

**FIGURE 4:**
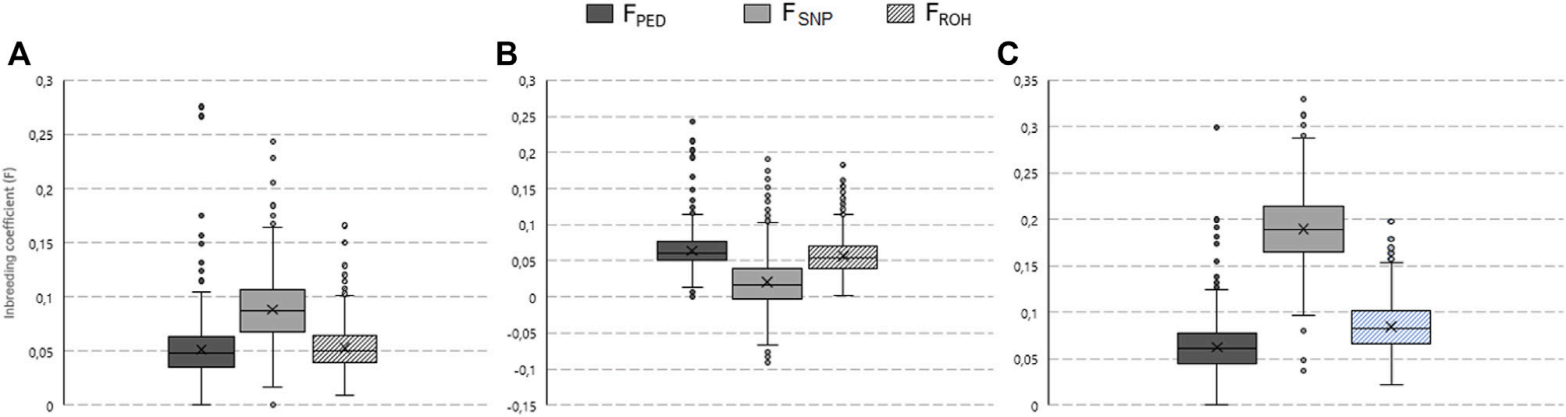
Box and whisker plots of the pedigree (F_PED_), single nucleotide polymorphism (F_SNP_), and runs of homozygosity (F_ROH_) based inbreeding coefficients estimated for the South African Ayrshire **(A)**, Holstein **(B)**, and Jersey **(C)** populations.

The variability in animal-specific inbreeding coefficients per breed for the genotyped animals is illustrated by box and whisker plots in Figure 4. Furthermore, a contingency table for pedigree *versus* genome-based estimates (i.e., F_SNP_ and F_ROH_) is included in Supplementary Material S2.

The mean F_PED_ for the AYR, HST, and JER genotyped populations was 0.051, 0.064, and 0.062, respectively. The highest frequency of AYR, HST, and JER animals fell within the 0.04-0.05, 0.05-0.06, and 0.07-0.08 coefficient classes, respectively. The greatest observed heterozygosity was in HST (H_O_ = 0.356) while the lowest was in JER (H_O_ = 0.332); hence, the F_SNP_-based inbreeding coefficient ranked the JER breed as the most inbred (F_SNP_ = 0.190) followed by the AYR (F_SNP_ = 0.088) and HST (F_SNP_ = 0.020) breeds. For the JER breed, for example, the majority of animals (1,323 animals of the population of 1,329 animals) were categorized as having high F_SNP_ values (>0.1) despite most of them having low (31.9% of animals) or moderate (62.7% of animals) F_PED_ values (Supplementary Material S2). The rank order of breeds (from largest to smallest mean) was different for the ROH-based inbreeding coefficients observed; F_ROH_ was the highest for the JER breed (mean F_ROH=_0.085), followed by the HST (mean F_ROH_ = 0.056) which was similar to the AYR (mean F_ROH_ = 0.053) breeds. The most AYR animals had F_ROH_ values in the 0.04 to 0.05 interval, whereas for HST and JER, most animals had F_ROH_ values of 0.05–0.06 and 0.06 to 0.07, respectively.

All F_ROH_ coefficients, irrespective of what length category was used to calculate the ROH, were highest for the JER population; the largest F_ROH_ statistic was obtained for F_ROH_ calculated for ROH that were larger than (or equal to) 4 Mb but smaller than 8 Mb ( $F_{{ROH}_{4\leq x<8Mb}}$ = 0.029). For $F_{{ROH}_{<4Mb}}$ and $F_{{ROH}_{4\leq x<8Mb}}$ , the AYR breed was similar in value to the HST breed (0.006 for AYR *versus* 0.004 for HST, and 0.019 for AYR *versus* 0.017 for HST, respectively), whereas HST had higher mean values for F_ROH_ calculated on the basis of longer ROH. For both the AYR and HST breeds, the $F_{{ROH}_{\geq 16Mb}}$ estimates were the highest (AYR: 0.019; HST: 0.022).

The Spearman correlations *(ρ*) between F_PED_ and genome-based F-statistics are given in Table 4. The correlation coefficients among all F statistics were strongest between the genome-based inbreeding estimates irrespective of the breed; the pairwise F_SNP_-F_ROH_ correlations ranged from *ρ* = 0.857 for AYR to *ρ* = 0.896 for JER. The F_PED_ coefficient was weak to moderately correlated with F_SNP_ and F_ROH_ within all breeds; the pairwise correlations between F_PED_ and each of the genome-based coefficients were similar (e.g., for AYR, *ρ* = 396 for both the F_PED_-F_SNP_ and F_PED_-F_ROH_ comparisons). The F_PED_ coefficient was most strongly related to $F_{{ROH}_{8\leq x<16Mb}}$ , compared to other F_ROH_ statistics, in both the AYR and HST breeds (*ρ* = 0.282, and *ρ* = 0.447, respectively); within the JER breed, however, the F_PED_ coefficient was most strongly related to $F_{{ROH}_{\geq 16Mb}}$ .

**TABLE 4:** Spearman correlations between the pedigree-based inbreeding coefficient (F_PED_) and various genomics-based inbreeding coefficients for the Ayrshire (AYR), Holstein (HST), and Jersey (JER) breeds.

		Genomic inbreeding coefficient					
		*F*_*SNP*_	*F*_*ROH*_	$F_{{ROH}_{<4Mb}}$	$F_{{ROH}_{4\leq x<8Mb}}$	$F_{{ROH}_{8\leq x<16Mb}}$	$F_{{ROH}_{\geq 16Mb}}$
F_PED_	AYR	0.396^**^	0.396^**^	0.132^**^	0.203^**^	0.282^**^	0.251^**^
	HST	0.584^**^	0.568^**^	0.284	0.425^**^	0.447^**^	0.406^**^
	JER	0.462^**^	0.455^**^	0.050	0.208^**^	0.310^**^	0.331^**^

$F_{{ROH}_{<4Mb}}$ , inbreeding coefficient based on runs of homozygosity (ROH) smaller than 4 Mb in size; $F_{{ROH}_{4\leq x<8Mb}}$ , inbreeding coefficient based on ROH larger and equal to 4 Mb but smaller than 8Mb; $F_{{ROH}_{8\leq x<16Mb}}$ , inbreeding coefficient based on ROH larger and equal to 8 Mb but smaller than 16Mb; $F_{{ROH}_{\geq 16Mb}}$ , inbreeding coefficient based on runs of homozygosity (ROH) larger than 16 Mb in size; ^**^ *p* < 0.001.

### 3.5 Effective population size

**FIGURE 5:**
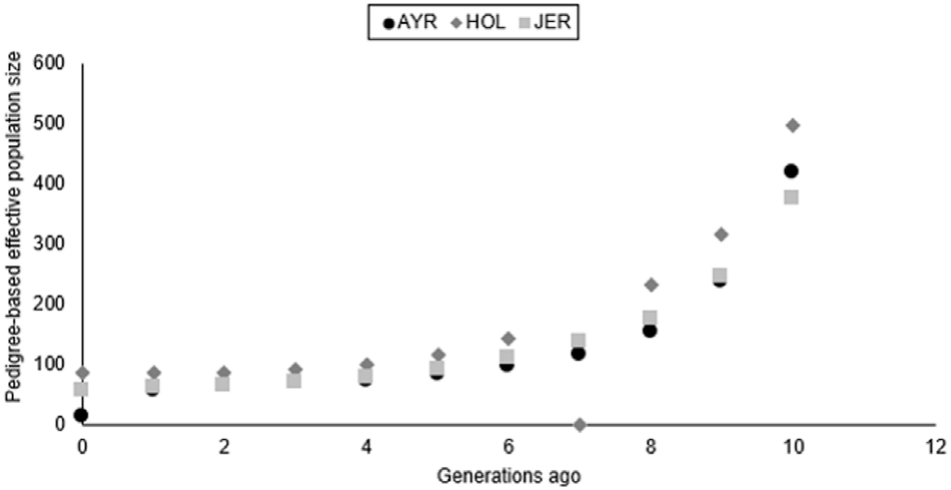
Pedigree-based estimates of effective population size (Ne) for the Ayrshire (AYR), Holstein (HST), and Jersey (JER) populations ten generations ago.

The pedigree-based Ne estimates increased for all three dairy cattle populations in this study (Figure 5) from 85 animals (generation 1) to 497 animals (generation 10) for HST, with a similar trend in the AYR and JER populations. The JER breed had the lowest pedigree-based estimates for the oldest (376 animals) and the youngest generation (57 animals). The AYR population experienced a large difference in Ne (362 animals) between generation 9 (419 animals) and generation 1 (57 animals). The most recent (12 generations ago) LD-based Ne was lowest for the AYR breed (Ne = 131) and the largest for JER (Ne = 149).

## 4 Discussion

To ensure sustainable breeding programs within the South African dairy industry, and to optimize the adoption of genome-based selection strategies, it is important to characterize and routinely monitor the genetic variability and inbreeding levels of the prominent dairy breeds (Howard et al., 2017). In the global dairy industry, strong directional selection, achieved by means of methodologies that favor the overuse of a few elite families (e.g., BLUP), as well as the application of advanced reproductive technologies (e.g., artificial insemination (AI)), has resulted in the accumulation of inbreeding, and hence, homozygosity (Maltecca et al., 2020). South Africa historically followed this trend of data-driven breeding programs, making use of international semen from a limited number of genetically elite bulls. In 2003, up to 36% of all dairy calves born in South Africa originated from foreign sires (Maiwashe et al., 2006). Due to the widespread use of AI, and easy access to phenotypic data and routine genotyping, the global dairy industry was the first livestock industry to embrace genome-wide enabled selection (GS) (Wiggans et al., 2011). However, in South Africa, genotyping on a commercial scale was only possible post-2016 for the dairy sector with the establishment of a DGP (Van Marle-Koster & Visser, 2018). This program was fundamental for the establishment of reference populations for the most popular dairy breeds used in the South African dairy industry (i.e., the AYR, HST, and JER breeds). The South African training populations remain small compared to many developed countries, but reflect the breed demographics on the national level and contain sufficient genotypes to assist in the genomic management of the populations. This study aimed to quantify homozygosity-based parameters of the AYR, HST, and JER populations in South Africa by using their pedigrees as well as 35,572 autosomal SNPs.

### 4.1 Pedigree completeness

Results from the present study indicate growing pedigree completeness over the past 10 generations with greater overall completeness in the AYR and JER populations. Traditionally, pedigree data has been used in the estimation of population diversity, but limitations on the quality and pedigree depth present limitations (the present study; Ablondi et al., 2022).

The high CGE calculated for the HST breed in this study (CGE = 11.70) aligns with previous studies of Canadian Holstein (CGE = 15.5, Stachowicz et al., 2011), Dutch Holstein (CGE = 12.5, Doekes et al., 2019), and Italian Holstein (CGE = 10.67, Ablondi et al., 2022). The JER population in this study (CGE = 10.05) is similar to that reported for Canadian Jerseys at 9.8 (Stachowicz et al., 2011) and higher than documented in Danish Jerseys (7.36; Sorensen et al., 2005). These studies, however, included data from animals born in earlier years when pedigree recording may not have been so ubiquitous. No literature was available on CGE for a genotyped AYR population. The lower CGEs for AYR and JER in the present study can be attributed to shallower pedigree depths in comparison to the HST breed due to CGE being dependent on the sum of the proportion of known ancestors over all generations traced (Wellmann and Bennewitz, 2019).

### 4.2 Within-breed genomic relatedness

Results of the autosomal SNP-based principal component analysis suggested a strong genetic influence of international bulls on the South African gene pool within all breeds. This result supports the fact that for all three of the studied breeds, the 25 most used AI sires (i.e., with the most daughters per breed with completed first lactations in 2021) were predominantly of international descent (SA Stud Book, 2022a; SA Stud Book, 2022b; SA Stud Book, 2022c). The greater observed heterozygosity in the HST population supported the more dispersed PCA clustering (and more outliers) and could be explained by the inclusion of more herds compared to the other breeds (1,360 animals from 411 herds for HST compared to only 510 genotyped animals from 31 herds for AYR), which would inevitably increase the extent of variation captured within the sampled population. The relatedness between a genomic selection reference population and the current (and active) population subjected to directional selection should be maintained and is integral to the accuracy of the produced genomic estimated breeding values (GEBVs) (Goddard & Hayes, 2009). Considering that genetic progress is directly related to, amongst other factors, the extent of genetic variation in a given population (Bourdon, 2000), the PCA-based results may serve as guidelines for future sampling and/or genotyping strategies to optimize genetic relatedness in genomic selection pipelines.

### 4.3 Runs of homozygosity detected

The profiling of genome-wide ROH has become an increasingly popular parameter for explaining genetic differences between populations; many ROH-based analyses have been conducted on global dairy breeds (e.g., Purfield et al., 2012; Mastrangelo et al., 2018; Doekes et al., 2020). Comparing these studies is, however, not trivial due to differences in the extent of genomic information available (higher density genotypes are expected to capture ROH profiles more comprehensively) and the methodologies used to detect ROH including the parameters specified when detecting an ROH (Gautason et al., 2021; Mulim et al., 2022). Interbreed differences in ROH number and length characteristics indicate historical differences between breeds within a certain country or region, or due to recent management actions (Xu et al., 2019). Although the HST and JER populations in the present study had similar numbers of individuals genotyped, 66% more ROH was detected in the JER (37,617 for JER compared to 22,674 for HST). The difference in the abundance of ROH segments relative to the AYR in the present study could have been influenced by the much smaller genotyped AYR population. Taking the sample sizes into account, the JER still had the most ROH per individual, but the AYR had more ROH per individual than the HST, irrespective of the detection method.

Despite the higher ROH counts observed for the JER population, the percentage genome coverage by autozygotic segments was the highest for the HST population (10.02%), followed by the AYR (8.80%) and JER (8.78%) populations. The percentage coverage was similar to the 10% reported by Kim et al. (2013) in US Holsteins and the 9.8% documented by Gautason et al. (2021) in Icelandic cattle. The higher proportion of large ROH segments (≥16 Mb) in the present study, representing inbreeding effects introduced up to ∼6 generations ago (Ferenčaković, 2015), observed for the HST population (CR: 0.079; SW: 0.109) implies a greater influence of more recent inbreeding in the population studied. Conversely, the higher proportion of short (<4 Mb in size) ROHs is indicative of older inbreeding effects and/or, possibly, recent admixture (and, hence, recombination) that could result in the breakdown of larger ROH (Purfield et al., 2012; Liu et al., 2021). Liu et al. (2021), for example, reported that ROHs as short as <1 Mb may be a result of ancestral inbreeding that occurred up to 50 generations ago; these related mating would be almost impossible to capture with pedigree information alone (especially considering the poor participation of South African dairy breeds in pedigree recording). It is clear that an analysis of ROH abundance and distribution can, therefore, be used to more comprehensively (and descriptively) explore genetic diversity within and between populations.

The percentage occurrence of SNPs residing within identified ROH was analyzed to identify overlapping genomic regions of autozygosity among animals within breeds. These overlapping regions could be the result of positive selection and could be indicative of adaptation to specific environmental conditions (Xu et al., 2019). The most frequent overlapping region identified in the present study was similar to those documented by Lozada-Soto et al. (2022) for North American dairy breeds; in agreement with Lozada-Soto et al. (2022), ROH hotspots (i.e., containing the highest SNP incidence within ROH segments) were also identified on BTA6 for AYR, BTA20 for HST, and BTA7 for JER. Two hotspots of homozygosity on BTA7 (base pairs: 41417884-42609605, and 42811272-45412030 base pairs) were in 70.96% and 70.88%, respectively, of the JER breed in the present study; these regions fall within the most gene-dense ROH island also documented for US Jersey (BTA7: 39.76-45.56Mb; Lozada-Soto et al., 2022).

Amongst the 35 protein-coding genes located in the first preserved region identified in the present study, is the *LYPD8* gene, which has been suggested to play a role in intestinal immunity in mice (Hsu et al., 2021) and more recently in sheep (Chen et al., 2022). The *HSPA4* gene, identified within the second most conserved region in the JER, is a heat shock protein (HSP) 70 gene and is well known for its integral role in cellular stress response to heat (Deb et al., 2014). Because of its lower body weight and, consequently, lower maintenance requirements, the JER breed is growing in popularity globally, especially given concerns over climate change and the expected increase in environmental stressors. The identification of conserved ROH segments containing genes, such as the prolactin receptor (*PRLR*) gene, highlights the higher selective pressure for milk productivity (Zhang et al., 2008) in HST. Regions overlapping with genes that are associated with heat stress and immune response (e.g., *PPEF2* for AYR as well as *ELANE*, and *LEAP2* for JER) further support the integral role that these breeds may play in the future sustainability of the South African dairy industry.

### 4.4 Inbreeding coefficients

As would be expected for dairy cattle populations, given factors such as the increased utilization of AI and other reproductive technologies compared to, for example, beef cattle, all inbreeding coefficients estimated in the present study suggested inbreeding is occurring. The F_PED_ values were generally lower than inbreeding estimates calculated from the genomic data. Perfect concordance was not expected between the F_PED_ values and those estimated using genomic information for several reasons including: 1) pedigree information will not always be complete all the way to the founder population, 2) the Meuwissen and Luo (1992) algorithm to estimate inbreeding assumes that animals in the pedigree with no recorded parents are unrelated and non-inbred, 3) pedigree errors undoubtedly exist (e.g., Sanarana et al., 2021), and 4) F_PED_ are based on expected relationships among individuals and cannot consider the variability that exists around this expectation owing to Mendelian sampling during gametogenesis (Kenny et al., 2023). The discrepancy between F_SNP_ and F_ROH_ may be attributed to the fact that F_SNP_ does not differentiate between alleles that are identical by descent (IBD) or identical by state (IBS) (Forutan et al., 2018) whereas F_ROH_ is influenced by, among other factors, the genome build (i.e., the reported position of each SNP relative to others) and SNP genotyping panel density. The suitability of each of the genomic measures is, therefore, dependent on the data available. The F_ROH_ coefficient is, however, more informative because of the additional information that the ROH length, for example, provides about the inbreeding history.

However, the means for F_PED_ (ranging from 0.051 for AYR to 0.064 for HST), as well as F_SNP_ (ranging from 0.02 for HST to 0.19 for JER) and F_ROH_ (ranging from 0.053 for AYR to 0.085 for JER), were similar in trend (albeit slightly lower in values) to those observed by Lozada-Soto et al. (2022) for North American dairy cattle; Lozada-Soto et al. (2022) reported F_PED_ means ranging from 0.06 for AYR to 0.08 for HST and JER, and F_ROH_ means ranging from 0.11 for AYR to 0.17 for JER. The effect of incomplete pedigree on the estimates of inbreeding is well documented (e.g., Lutaaya et al., 1999; Marshall et al., 2002; Cassell et al., 2003), and it is generally accepted that incomplete and inaccurate pedigree recording leads to an underestimation of pedigree-based inbreeding coefficients. Tested against F_PED_ per breed, the Spearman correlations with both F_SNP_ and length-specific F_ROH_ estimates were weak to moderate and slightly weaker than, but comparable to, those reported by, for example, Gautason et al. (2021) using a 50,000 SNP genotyping panel on over 8,000 Icelandic cattle (*ρ* for F_PED_-F_IS_ = 0.52; *ρ* for F_PED_-F_ROH_ = 0.63). Cortes-Hernández et al. (2021) observed similarly weak correlations between F_PED_ and genome-based coefficients (e.g., 0.39 with F_SNP_ and 0.30 with F_ROH_) in a small Mexican Holstein population genotyped for 100,806 SNPs. Nonetheless, the pairwise correlations between F_PED_ and F_ROH_ improved as ROH length increased. Irrespective of breed, the correlation between F_PED_ and F_ROH<4MB_ was the weakest of all correlations between F_PED_ and length-specific F_ROH_ coefficients. This observation agrees with previous suggestions that correlations between F_PED_ and F_ROH_ strengthen when the shortest ROH fragments (typically those less than 4 Mb) are not considered in the calculation (Purfield et al., 2012). The phenomenon of a strengthening correlation between F_PED_ and F_ROH_ as ROH length increases suggests that the relationship between F_PED_ and F_ROH_ is probably influenced by the breed-specific pedigree depth (Cortes-Hernández et al., 2021). Many previous studies have reported stronger F_PED_-F_ROH_ correlations for populations with deeper recorded pedigree (e.g., Purfield et al., 2012; Ferenčaković, 2015; Peripolli et al., 2018), as was the case with the HST (pedigree depth = 11.70; *ρ* for F_PED_-F_ROH≥16Mb_ = 0.406) compared to AYR (pedigree depth = 9.75; *ρ* for F_PED_-F_ROH≥16Mb_ = 0.251). Considering the generally low within-breed participation in pedigree recording for South African dairy breeds (as low as 24%; Van Marle-Köster & Visser, 2018), the accuracy of pedigree-based inbreeding coefficients (and by extension relationships between individuals) should be interpreted with caution.

### 4.5 Effective population size

Factors that influence Ne estimates include the constant change in the real population size, unequal sex ratios, and the variance in the number of offspring per parent (Nielsen and Slatkin, 2013). A reduction in Ne in livestock is generally the consequence of selection pressure on traits of economic importance, exacerbated by the use of a few high-impact sires *via* reproductive technologies (Mulim et al., 2022). The pedigree-based Ne estimates of the youngest animals in the present study all exceed the FAO guideline of 50 animals (OECD-FAO, 2019) but it must be noted that they have all reduced substantially over the last 10 generations. Canadian, Danish, Dutch, Irish, Italian, and US HST populations have reported pedigree-based Ne of the youngest generation to be 39, 70, 49, 75, and 39 (Weigel, 2001; Sorensen et al., 2005; McParland et al., 2007; Makanjuola et al., 2020; Ablondi et al., 2022) animals, respectively. The South African HST population had the highest Ne (i.e., 85) of the three South African dairy breeds investigated in the present study which may be a consequence of the greater completeness of the pedigree used and/or the use of a larger number of genetically dissimilar sires sourced from multiple countries. Sorensen et al. (2005) reported the pedigree-based Ne of Danish Jersey cattle to be 116 while Stachowicz et al. (2011) reported a pedigree-based Ne of 54 for Canadian Jersey. A more recent study on Canadian Jersey cattle populations suggested an Ne of 49 animals (Makanjuola et al., 2020). Estimates of Ne for the South African Jersey yielded a similar low of 57 animals, as well as South African AYR with a Ne of 57 nine generations ago which points to lower genetic diversity within these two breeds in comparison to the HST breed. Although previously reported Ne estimates vary widely amongst populations, Brotherstone & Goddard (2005) reported that the Ne of most modern dairy cattle populations is circa. 100. The predictions for the South African dairy populations are also between 50 and 100 animals. Because of the hyperbolic relationship between LD (*r* ^2^) and Ne, more recent (i.e., fewer generations ago), estimates of genome-based Ne are possible with a greater density of SNPs and, therefore, is better at capturing population-wide LD (Barbato et al., 2015). Genomic optimum contribution selection may be a viable tool for dairy breeding programs as it will increase genetic merit while maintaining genetic diversity (Clark et al., 2013). Genetic gain of South African dairy breeds may increase due to the current use of GEBVs (Van der Westhuizen and Mostert, 2020) and will aid in minimizing the loss of fitness by preventing any further reduction in Ne. Although the current Ne rates indicate that inbreeding is well-managed, it should still be monitored regularly to avoid adverse effects in future generations.

## 5 Conclusion

The South African AYR has always been a small population serving a niche market, while the South African HST and JER breeds are mainly responsible for the fresh milk supply. It will be important for these breeds to grow and maintain their reference populations and ensure that international bull families and genotypes are available for genetic evaluations and continuous monitoring of diversity and inbreeding. This study confirmed the usefulness of SNP genotypes for accurately assessing autozygosity and inbreeding levels, and the impact of these on the management of genetic resources. The analyzed results support the influence of globalized dairy germplasm and their observed influences on the genetic diversity within the JER, HST, and AYR reference populations in South Africa thus far. Since the erosion of genetic diversity limits long-term genetic gain and impedes resilience and sustainability amidst future challenges, these results may assist in strategies to improve and update reference populations for genomic selection.

## Data Availability

The data analyzed in this study is subject to the following licenses/restrictions: Genomic data was obtained from SA Stud Book, a local service provider of genetic and genomic analyses in South Africa. The data thus belongs to third parties (SA Stud Book and Breed Societies). Requests to access these datasets should be directed to the corresponding authors.

## Supplementary material

Supplementary Material S1 A figure showing the number of run of homozygosity (ROH) per chromosome on the primary axis (bar graphics), as well as the chromsome-wise ROH coverage on the secondary axis (line graphs), for the Ayrshire (AYR), Holstein (HST), AND Jersey (JER) population.

Supplementary Material S2 Contingency tables of the frequency of animals within different classes of pedigree-based inbreeding coefficients (F_PED_) that fell within three categories (low, medium, and high) of genomics-based inbreeding coefficients (F_SNP_, and F_ROH_) for the Ayrshire (AYR), Holstein (HST), and Jersey (JER) breeds.
