# Supplementary material for: Pedigree and genome-based patterns of homozygosity in the South African Ayrshire, Holstein, and Jersey breeds
Source: Front Genet. 2023 Mar 17;14:1136078. doi: 10.3389/fgene.2023.1136078 (PMC10063850; doi:10.3389/fgene.2023.1136078)
Supplement: Supplementary file 1 [file Table1.pdf]

Supplementary Material 2 | Contingency tables of the frequency of animals within different classes of pedigree-based inbreeding coefficients (F_{PED}) that fell within three categories (low, medium, and high) of genomics-based inbreeding coefficients (F_{SNP}, and F_{ROH}) for the Ayrshire (AYR), Holstein (HST), and Jersey (JER) breeds.

		F_{SNP}			F_{ROH}		
**Population**	**F_{PED} class**	Low	Moderate	High	Low	Moderate	High
**AYR**	0.000-0.050	160	20	89	134	126	9
	0.050-0.100	26	134	67	107	112	8
	>0.100	0	10	4	10	3	1
	**Total**	**186**	**164**	**160**	**251**	**241**	**18**
**HST**	0.000-0.050	293	39	4	153	173	10
	0.050-0.100	762	129	29	408	471	41
	>0.100	63	26	10	20	68	11
	**Total**	**1,118**	**194**	**43**	**581**	**712**	**62**
**JER**	0.000-0.050	0	1	422	31	276	116
	0.050-0.100	2	2	829	76	528	229
	>0.100	0	1	72	9	44	20
	**Total**	**2**	**4**	**1,323**	**116**	**848**	**365**
