# Supplementary figures and images for: Pedigree and genome-based patterns of homozygosity in the South African Ayrshire, Holstein, and Jersey breeds
Source: Front Genet. 2023 Mar 17;14:1136078. doi: 10.3389/fgene.2023.1136078 (PMC10063850; doi:10.3389/fgene.2023.1136078)

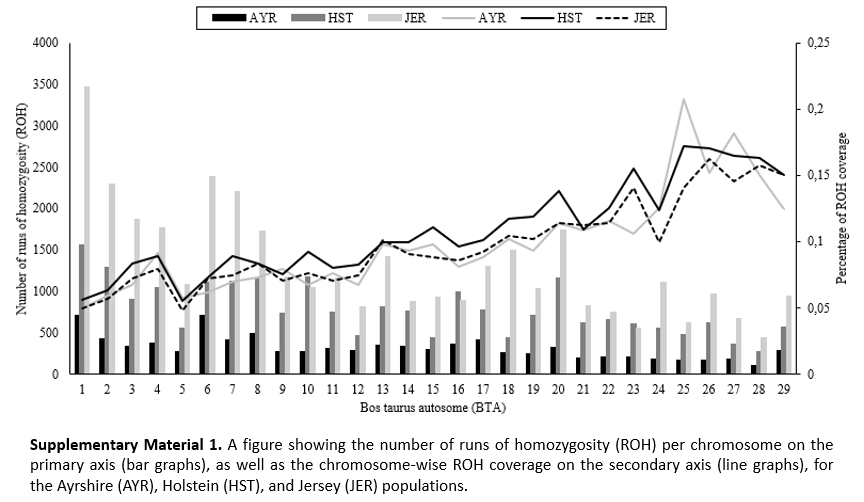

Supplement: Supplementary file 2 [file Image1.tif]
